# Neutralizing S1P inhibits intratumoral hypoxia, induces vascular remodelling and sensitizes to chemotherapy in prostate cancer

**DOI:** 10.18632/oncotarget.3144

**Published:** 2015-01-29

**Authors:** Isabelle Ader, Cécile Gstalder, Pierre Bouquerel, Muriel Golzio, Guillaume Andrieu, Santiago Zalvidea, Sylvain Richard, Roger A. Sabbadini, Bernard Malavaud, Olivier Cuvillier

**Affiliations:** ^1^ CNRS, Institut de Pharmacologie et de Biologie Structurale, Toulouse, France; ^2^ Université de Toulouse, UPS, IPBS, Toulouse, France; ^3^ Equipe Labellisée Ligue Contre le Cancer, Toulouse, France; ^4^ Hôpital Rangueil, Service d'Urologie et de Transplantation Rénale, Toulouse, France; ^5^ INSERM U1046, Université Montpellier 1, Université Montpellier 2, CHU Arnaud de Villeneuve, Montpellier, France; ^6^ Lpath Inc., San Diego, CA, USA

**Keywords:** angiogenesis, hypoxia, sphingolipid, vessel normalization

## Abstract

Hypoxia promotes neovascularization, increased tumor growth, and therapeutic resistance. The transcription factor, hypoxia-inducible factor 1α (HIF-1α), has been reported as the master driver of adaptation to hypoxia. We previously identified the sphingosine kinase 1/sphingosine 1-phosphate (SphK1/S1P) pathway as a new modulator of HIF-1α under hypoxia. Taking advantage of a monoclonal antibody neutralizing extracellular S1P (sphingomab), we report that inhibition of S1P extracellular signaling blocks HIF-1α accumulation and activity in several cancer cell models exposed to hypoxia. In an orthotopic xenograft model of prostate cancer, we show that sphingomab reduces hypoxia and modifies vessel architecture within 5 days of treatment, leading to increased intratumoral blood perfusion. Supporting the notion that a transient vascular normalization of tumor vessels is the mechanism by which sphingomab exerts its effects, we demonstrate that administration of the antibody for 5 days before chemotherapy is more effective at local tumor control and metastatic dissemination than any other treatment scheduling. These findings validate sphingomab as a potential new normalization agent that could contribute to successful sensitization of hypoxic tumors to chemotherapy.

## INTRODUCTION

Sphingosine 1-phosphate (S1P) is a bioactive sphingolipid metabolite regulating pleiotropic activities such as proliferation, survival, migration, inflammation or angiogenesis [1–4]. The S1P content in cells is low and is kept under control through a delicately regulated balance between its synthesis and its degradation. The balance between the intracellular levels of S1P and its metabolic precursors, ceramide and sphingosine, has been suggested to be a switch determining whether a cell proliferates or dies [5]. The predominant regulator of this ceramide/S1P balance is the sphingosine kinase-1 (SphK1) isoform, which produces S1P from sphingosine [6]. Once generated, S1P is exported by specific transporters such as spinster 2 (Spns2) [7–9], to exert paracrine or autocrine effects as a ligand for five high-affinity G protein-coupled receptors (S1P_1–5_), with specific effects dictated by the predominance of S1P receptor subtypes expressed [10]. Alternative GPCR-independent signaling of S1P also exist [11] with recent findings demonstrating direct modulation of several intracellular proteins [12, 13].

In cancer, S1P metabolism is often found to be dysregulated directing attention to the SphK1/S1P signaling pathway as a target for anti-cancer drug discovery [14–16]. In a number of solid tumors, high SphK1 expression correlates with a significant decrease in survival rate in patients [17–20]. The S1P produced by SphK1 is widely appreciated as a general growth-like factor and a potent protector against apoptosis induced by cytotoxic agents and other therapies in various cancer cell and animal models [14]. S1P is also thought to be as pro-angiogenic as basic fibroblast growth factor and Vascular Endothelial Growth Factor (VEGF) in promoting the development of vascular networks *in vivo* [21–24]. A number of preclinical studies have shown that pharmacological inhibition of SphK1 could be efficacious in decreasing tumor size or sensitize to chemo- or radiotherapy [25–28]. Interestingly, the anti-cancer activity of an anti-S1P monoclonal antibody (sphingomab™) [29], which neutralizes S1P and inhibits its extracellular signaling, provides evidence of the importance of exogenous S1P in mediating tumor growth and metastatic potential [23, 30, 31].

Hypoxia is a reduction in the normal level of tissue oxygen tension and occurs in many pathological conditions including cancer [32] where it contributes to the development of an aggressive phenotype and a poor prognostic in patients [33]. As a tumor develops, the diffusion distance from the existing vasculature increases resulting in hypoxia, which in turn drives the overexpression of angiogenic factors such as VEGF, leading to the formation of a new vasculature in an attempt to provide adequate supply of oxygen and nutriments [34, 35]. Somewhat paradoxically, such unleashed angiogenesis generates a highly disorganized and immature vascular network with impaired transport characteristics resulting in spatial and temporal inadequacies in delivery of oxygen, thereby exacerbating tumor hypoxia and fuelling a self-reinforcing vicious cycle [36, 37]. As a result of the leakiness of tumor vessels, impaired blood flow and interstitial hypertension interfere with the delivery of therapeutics reducing their efficacy while promoting the escape of cancer cells [37–41]. At the cellular level, the activation of the transcription factor hypoxia-inducible factor 1 (HIF-1) [42], has been identified as a master regulator of the response of cancer cells to hypoxia, triggering the expression of multiple target genes contributing to angiogenesis, treatment failure, invasion/metastasis, altered metabolism and genomic instability [32, 43].

Given its central role in tumor progression and resistance to therapy, targeting hypoxia-induced angiogenesis represent an attractive strategy in cancer centered on two molecular targets, HIF-1 and VEGF [44–46]. As the direct inhibition of a transcription factor is a challenging task [47], targeting upstream signaling pathways leading to HIF-1 activation or downstream effectors regulated by HIF-1 such as VEGF may represent a more practical strategy and a wide range of pharmacological approaches have been proposed including the targeting of the SphK1/S1P signaling [48, 49]. Indeed, we previously identified SphK1/S1P signaling as a new canonical modulator of HIF-1 activity under hypoxic conditions owing to a decreased proteasome degradation of HIF-1α subunit mediated by the Akt/GSK3β pathway in various cancer cell models [50]. Because Akt signaling can be activated by Gi-coupling of all subtypes of S1P receptors [10] and because S1P has been shown to be released from hypoxic cells [51, 52], we have explored the effects of the neutralization of extracellular S1P with anti-S1P monoclonal antibody sphingomab, currently under clinical development [15]. The goal of this study was to demonstrate preclinical proof of concept in mice bearing orthotopic prostate tumors that sphingomab could reduce intratumoral hypoxia and associated vascular network malfunction by enhancing blood perfusion to significantly improve delivery and efficacy of docetaxel, the standard chemotherapy for prostate cancer.

## RESULTS

### Extracellular S1P regulates HIF-1α level under hypoxia in several cancer cell lineages

**Figure 1:**
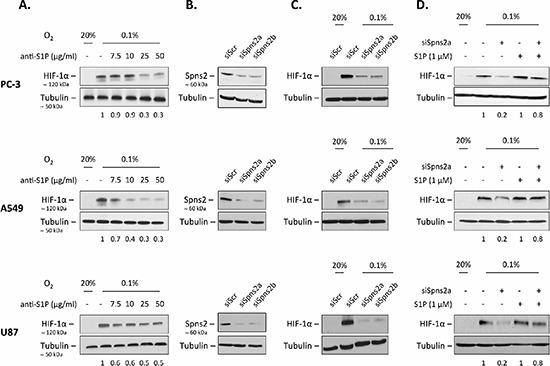
Extracellular S1P regulates HIF-1α level under hypoxia **(A)** Human PC-3, A549 and U87 cells were treated with the indicated concentrations of anti-S1P mAb (anti-S1P) for 2 h, then incubated under normoxia (20% O_2_) or hypoxia (0.1% O_2_) for an additional 6 h. HIF-1α expression was analyzed by immunoblotting using an anti-HIF-1α antibody. **(B)** PC-3, A549 and U87 cells were untransfected or transfected with 90 nmol/L of two different siSpns2 (siSpns2a and siSpns2b) or scrambled siRNA (siScr) for 72 h. Cell lysates were assayed for Spns2 expression by Western blot. **(C)** PC-3, A549 and U87 cells were untransfected or transfected with 90 nmol/L of two different siSpns2 (siSpns2a) and siSpns2b) or scrambled siRNA (siScr) for 72 h, then incubated under normoxia or hypoxia for an additional 6 h. Cell lysates were assayed for HIF-1α expression by Western blot. **(D)** PC-3, A549 and U87 cells were untransfected or transfected with 90 nmol/L of siSpns2a for 72 h. The cells were then washed and left in DMEM without serum for 12 h before the incubation under normoxia or hypoxia without or with 1 μM S1P for an additional 6 h. Cells were lysed and HIF-1α expression was analyzed by immunoblotting with an anti-HIF-1α antibody. For all experiments, similar results were obtained in at least three independent experiments, and equal loading was monitored using antibody to α-tubulin.

We previously identified SphK1 as a modulator of HIF-1α as a key mediator of the adaptive response to hypoxia in multiple cancer cell models [50]. These studies led us to propose a strategy for controlling tumor hypoxia and its biological consequences [48]. To substantiate that inhibition of the SphK1/S1P pathway could represent a pertinent idea, we evaluated the relevance of inhibiting the extracellular S1P signaling with regard to HIF-1α accumulation under hypoxia in cancer cells. We took advantage of a monoclonal antibody (mAb), sphingomab, that binds to and neutralizes extracellular S1P [23, 29]. As shown in Figure 1A, sphingomab inhibited accumulation of HIF-1α in a concentration-dependent manner in human PC-3 prostate cancer cells. The ability of the anti-S1P mAb to inhibit HIF-1α accumulation was tested in two other models, including the lung adenocarcinoma cell line A549, and the glioblastoma cell line U87. A similar dose- dependent action of the anti-S1P mAb on HIF-1α content was observed in these models (Figure 1A). S1P is mainly produced intracellularly by SphK1 and exerts its paracrine/autocrine effects by being secreted into the tumor microenvironment. Spinster 2 (Spns2) has been recently suggested to be the primary transporter in the release of S1P [7–9, 53, 54]. When PC-3, A549 and U87 cells were treated with Spns2-specific siRNAs, the expression of Spns2 protein decreased to less than 10–20% of the control with two different siRNAs tested (siSpns2a and siSpns2b) (Figure 1B). The transient knockdown of Spns2 using these two different siRNAs was associated with a significant inhibitory effect on HIF-1α accumulation under hypoxia (Figure 1C). Importantly, the decrease of HIF-1α protein content by Spns2 targeting was markedly reversed when cells were exposed to S1P (Figure 1D). These results agree with the data in Figure 1A and demonstrate that extracellular S1P released from cancer cells is critical to regulate HIF-1α accumulation under hypoxia, regardless of the experimental conditions (10% FBS in Figure 1A *versus* serum free conditions in Figure 1D).

### Neutralization of extracellular S1P downregulates HIF-1α expression and activity *in vivo*

**Figure 2:**
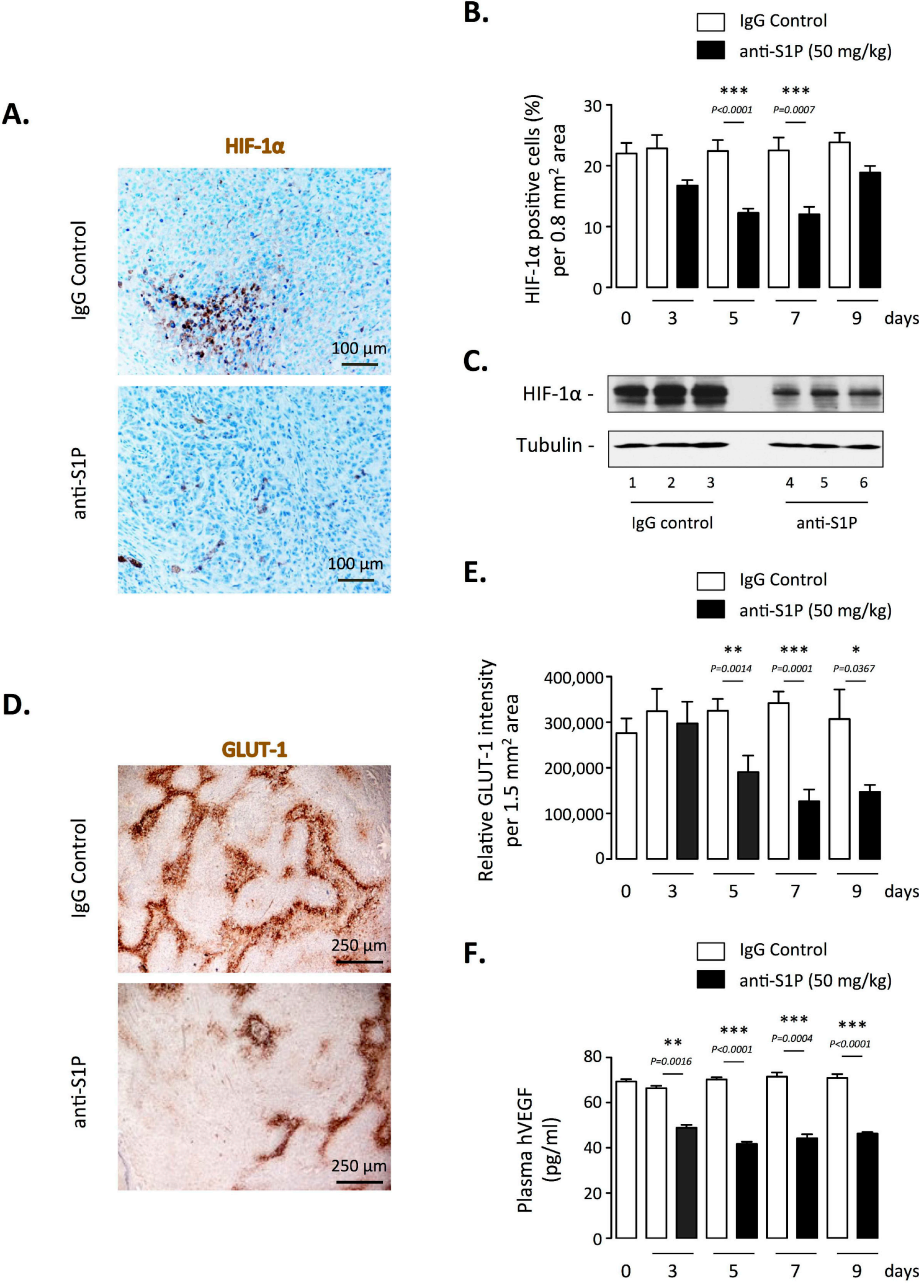
Effect of S1P neutralization on HIF-1α expression and activity *in vivo* Nude mice were injected in prostate with PC-3/GFP cells to form orthotopic xenografts. 21 days after injection, mice were treated every odd day with 50 mg/kg anti-S1P mAb or IgG control. After 3, 5, 7 and 9 days of treatment, mice were sacrificed and the primary tumors and blood were collected. **(A)** HIF-1α staining of representative regions of tumor sections from animals treated for 5 days with 50 mg/kg anti-S1P antibody or IgG control. Scale bar, 100 μM. **(B)** Graph represents percentage of HIF-1α positive cells per 0.8 mm2 area after 3, 5, 7 and 9 days of treatment. *Columns*, mean of four mice per group; *bars*, SEM. **(C)** Analysis of HIF-1α protein expression by Western blotting in tumor lysates harvested from three mice per group after 5 days of treatment with IgG control (lane 1–3) or anti-S1P (lane 4–6). α-tubulin was used as loading control. **(D)** GLUT-1 staining of representative regions of tumor sections from animals treated for 5 days with 50 mg/kg anti-S1P mAb or IgG control. Scale bar, 250 μM. **(E)** Graph represents relative GLUT-1 intensity per 1.5 mm^2^ area after 3, 5, 7 and 9 days of treatment. *Columns*, mean of four mice per group; *bars*, SEM. **(F)** Plasma hVEGF concentrations in tumor-bearing mice after 3, 5, 7 and 9 days of treatment with 50 mg/kg anti-S1P antibody or IgG control. *Columns*, mean of four mice per group; *bars*, SEM.

To extend our *in vitro* findings, and because the anti-S1P mAb we used is under clinical development, the antibody's ability to inhibit HIF-1α accumulation and transcriptional activity *in vivo* was tested in an orthotopic model of prostate cancer using the PC-3 cell line, because hypoxic response is largely dependent on the vascular microenvironment and very poorly replicated in subcutaneous models [55]. As previously reported [25, 56], 21 days after implantation of human GFP-overexpressing PC-3 in nude mice, the histology of the tumor was consistent with poorly differentiated prostate cancer (data not shown) and animals were treated every other day for up to 9 days with 50 mg/kg sphingomab or the isotype-matched IgG control by i.p. administration. Blood and primary tumors were collected from animals sacrificed at Day 0 or after 3, 5, 7 and 9 days of treatment with anti-S1P or IgG control antibodies. The excised tumors were first evaluated for immunohistochemical analysis of HIF-1α expression. As shown in Figure 2A, anti-HIF-1α-labeled sections showed a marked decrease in staining of tumor cells in mice treated for 5 days with the anti-S1P mAb. A highly significant reduction of the number of HIF-1α-positive cells in tumor sections was observed from 5 to 7 days of treatment (*P* < 0.0001) but returned to the control value at 9 days suggesting a transient effect of the anti-S1P mAb (Figure 2B). Western blot analysis on tumor lysates collected at Day 5 confirmed the marked decrease of HIF-1α protein expression when animals were treated with anti-S1P mAb in all samples examined (Figure 2C).

GLUT-1-stained sections revealed that inhibitory effect of the anti-S1P mAb was not restricted to HIF-1α protein accumulation but was also correlated with an inhibition of its transcriptional activity (Figure 2D). The treatment with anti-S1P mAb led to more than 50% inhibition of GLUT-1 expression as early as 5 days of treatment up to 9 days with a maximal effect seen at Day 7 (Figure 2E). In addition, animals treated with the anti-S1P mAb had significantly reduced levels of circulating proangiogenic human VEGF (hVEGF) produced by the PC-3 xenografts (Figure 2F). Collectively these *in vivo* data demonstrate that S1P neutralization not only downregulates HIF-1α content but also decreases the expression of GLUT-1 and the secretion of VEGF, two main factors depending on HIF-1α transcriptional activity.

### Neutralization of S1P is associated with decreased intratumoral hypoxia, vascular remodelling and increased blood flow

**Figure 3:**
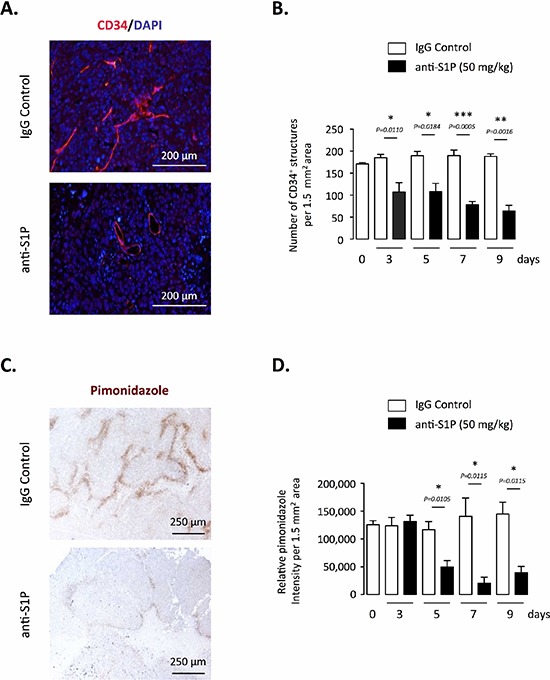
Effect of S1P neutralization on tumor vasculature and hypoxia in PC-3 tumor-bearing mice **(A)** Representative images of blood vessel quantity and morphology in tumors after 5 days of treatment with 50 mg/kg anti-S1P mAb or IgG control. Tissue sections were stained with anti-CD34 (endothelial cell marker) antibody to visualize tumor blood vessel and were counterstained with DAPI. Scale bar, 200 μM. **(B)** Data are shown as number of CD34^+^ structures per 1.5 mm^2^. *Columns,* mean of four mice per group; *bars*, SEM. **(C)** Hypoxia staining of representative regions of tumor sections from animals treated for 5 days with 50 mg/kg anti-S1P antibody or IgG control. Hypoxia was detected by immunohistochemistry staining of pimonidazole adducts in PC-3 tumor sections. Nuclear counterstain: hematoxylin staining. Scale bar, 250 μM. **(D)** Graph represents relative HypoxiaProbe intensity after 3, 5, 7 and 9 days of treatment with anti-S1P mAb or IgG control. *Columns,* mean of four mice per group; *bars*, SEM.

We previously observed that neutralization of S1P was associated with a reduction in the release of the potent vascular permeability factor VEGF from tumors, validating the sphingomab as an antiangiogenic agent [23]. We therefore analyzed effects of the antibody on the vasculature of orthotopic PC-3 tumor using immunohistochemical expression of CD34 as biological marker of angiogenesis. In agreement with the *in vivo* effect formerly reported in a subcutaneous A549 model [23], immunohistochemical analysis indicated a marked decrease in microvessel density in mice treated with the anti-S1P mAb throughout all the experiment time course (Figure 3A and 3B). The remaining vessels seemed to be opened indicating that “immature” vessels were likely preferentially eliminated (Figure 3A). The treatment with the anti-S1P mAb was accompanied by a marked reduction of intratumoral hypoxia as measured by pimonidazole staining, an effect that was significant as early as 5 days of treatment with a persisting effect that peaked at Day 7 (Figure 3C and 3D).

**Figure 4:**
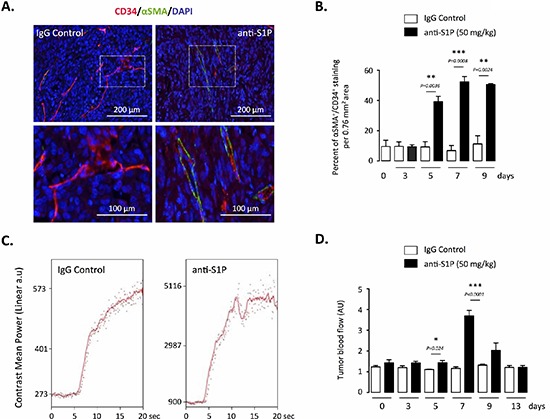
Effect of S1P neutralization on vessel functionality **(A)** Representative images of immunofluorescence double staining for endothelial cells (CD34) and pericytes (αSMA) in paraffin sections obtained from tumors of animals treated with 50 mg/kg anti-S1P mAb or IgG control. Red, CD34^+^ staining; green, αSMA^+^ staining. Counterstaining was done with DAPI. Images were obtained from mice 7 days after beginning of the treatment. Scale bar, 200 μM. **(B)** Graph represents quantification of vessel coverage per 0.76 mm^2^ area, calculated as the percentage of αSMA-positive cells compared with the number of CD34-positive cells after 3, 5, 7 and 9 days of treatment with 50 mg/kg anti-S1P mAb or IgG control. *Columns,* mean of four mice per group; *bars*, SEM. **(C)** Study of tumor vessel perfusion by contrast-enhanced ultrasound imaging. Examples of raw contrast kinetics acquired after bolus i.v. injection of microbubble contrast agent in animals treated with 50 mg/kg IgG control or anti-S1P mAb for 7 days. **(D)** Quantification of microbubble velocity within o.t PC-3 tumors, after 3, 5, 7, 9 and 13 days of treatment with anti-S1P mAb or IgG control. *Columns,* mean of four mice per group; *bars*, SEM.

We next determined how hypoxia and neoangiogenesis were antagonized by the anti-S1P mAb. We first evaluated the architecture and the functional status of tumor vasculature. Because perivascular pericytes play a critical role in vessel maturation and stabilization [37], a double staining for pericytes (α-smooth muscle actin or α-SMA) and endothelial cells (CD34) was performed to quantify the extent of pericyte coverage. A significant increase of the number of α-SMA-positive intratumoral blood vessels was found in tumor-bearing animals treated with anti-S1P mAb, demonstrating the characteristic development of more mature vessels (Figure 4A and 4B). As noticed for VEGF level and intratumoral hypoxia markers, the effect of sphingomab was evident after 5 days of treatment and maintained overtime (up to 9 days). To examine whether the morphological vessel normalization obtained by neutralizing S1P could translate into a functional vasculature, blood perfusion was assessed by a non-invasive real-time imaging using high frequency ultrasound to allow for longitudinal study on the same animal [57, 58]. At different times after administration of anti-S1P mAb or control IgG, the slope and magnitude of microbubble contrast agent influx was measured to quantify the blood flow in vessels (Figure 4C and 4D). In anti-S1P mAb-treated mice, tumor perfusion significantly augmented as early as 5 days of administration, peaked at Day 7 (over 3-fold increase), then returned to basal level by Day 13 (Figure 4C and 4D). Collectively, these results showing the reduction in tumor hypoxia and enhanced vascular flow when S1P is neutralized support the notion that a transient “vascular normalization” [41] is the mechanism by which anti-S1P mAb exerts its effects, an effect which could enhance the efficacy of cytotoxic agents (see below).

### Anti-S1P mAb's direct effects on tumor cell proliferation and apoptosis

**Figure 5:**
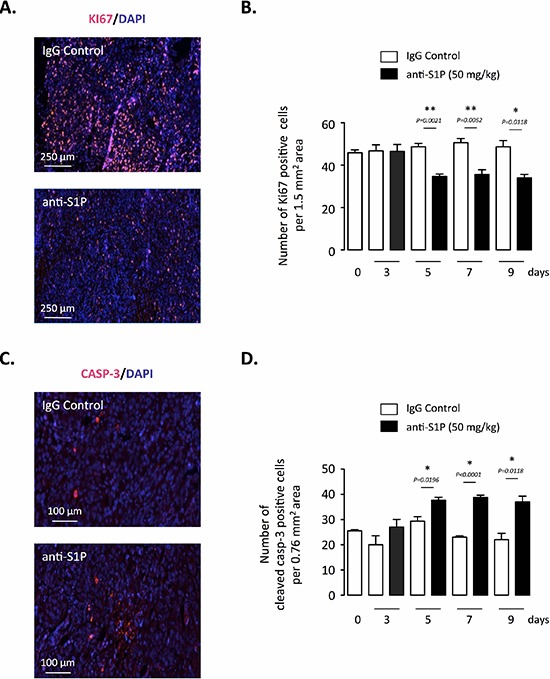
Effect of S1P neutralization on tumor cell proliferation and apoptosis of established orthotopic PC-3/GFP tumors in mice **(A)** Ki67 staining of representative tumor sections after 7 days of treatment with 50 mg/kg anti-S1P mAb or IgG control. Red, Ki-67 positive staining. Counterstaining was done with DAPI. **(B)** Data are shown as number of Ki67-positive cells per 1.5 mm^2^ area after 3, 5, 7 and 9 days of treatment with anti-S1P mAb or IgG control. *Columns,* mean of four mice per group; *bars*, SEM. **(C)** Cleaved caspase-3 (CASP-3) staining of representative tumor sections after 7 days of treatment with 50 mg/kg anti-S1P mAb or IgG control. Red, cleaved caspase-3-positive staining. Counterstaining was done with DAPI. **(D)** Data are shown as number of cleaved caspase-3-positive cells per 0.76 mm^2^ area after 3, 5, 7 and 9 days of treatment with anti-S1P mAb or IgG control. *Columns,* mean of four mice per group; *bars*, SEM.

S1P is a well-established proliferative and anti-apoptotic mediator [5, 59], the ability of anti-S1P antibody to alter cell proliferation and death of PC-3 xenograft was also evaluated. The Ki67 proliferation marker was used to estimate the fraction of viable cells undergoing active proliferation within the tumor. Anti-S1P mAb-treated tumors exhibited a notable decrease in cell proliferation rate that was evident after 5 days of administration (Figure 5A and 5B). Tumor cell death was assessed by the activation of the apoptotic executioner caspase-3. In agreement with previous reports showing that S1P inhibits activation of caspase-3 [23, 60], the treatment with anti-S1P mAb was associated with increased apoptosis as reflected by the increased level of cleaved caspase-3 (Figure 5C and 5D). These data suggest that the proliferative and protective antiapoptotic effects of S1P are mitigated when extracellular S1P is neutralized by the anti-S1P antibody.

### Anti-S1P mAb-induced vascular normalization sensitizes to chemotherapy in established orthotopic PC-3/GFP tumors in mice

**Figure 6:**
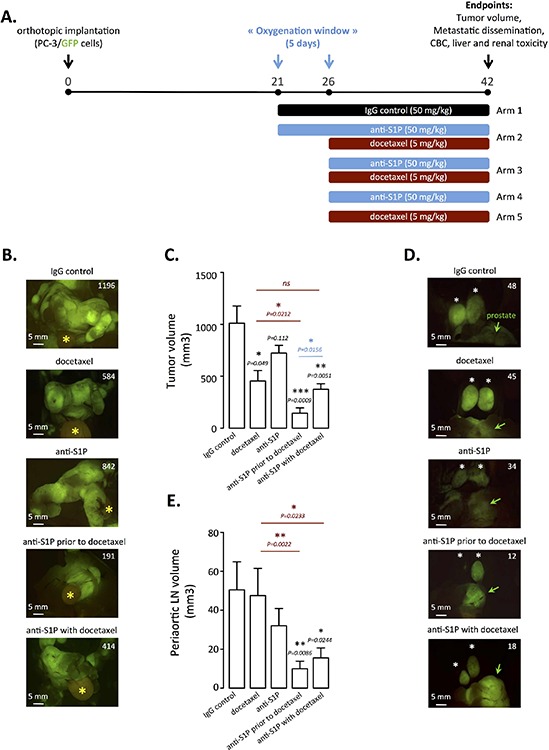
Docetaxel treatment enhancement by rational scheduling of S1P neutralization on established orthotopic PC-3/GFP tumors in mice **(A)** Three weeks after surgical orthotopic implantation of PC-3/GFP cells, mice were randomized into five arms of three to seven animals each. These animals were then subjected to 50 mg/kg IgG control every odd day (arm 1, *n* = 4); 50 mg/kg anti-S1P mAb every odd day prior to weekly 5mg/kg docetaxel starting at day 26 (arm 2, *n* = 6); combination of 50 mg/kg anti-S1P mAb every odd day and weekly 5mg/kg docetaxel starting at day 26 (arm 3, *n* = 5); 50 mg/kg anti-S1P mAb every odd day starting at day 26 (arm 4, *n* = 7); weekly 5 mg/kg docetaxel starting at day 26 (arm 5, *n* = 3). At day 42, mice were anesthetized and tumor volume, metastatic dissemination were determined by fluorescence imaging and blood was taken for complete blood count (CBC) and plasma chemistry. **(B)** Representative fluorescent primary PC-3/GFP tumors from animals treated with IgG control, docetaxel, anti-S1P mAb, anti-S1P mAb prior to docetaxel and combination of docetaxel and anti-S1P mAb, at the time of autopsy. Yellow asterisk indicates bladder. Numbers represent tumor volume as quantified in materials and methods. **(C)** Quantification of tumor volume of primary tumors. *Columns,* mean of three to seven mice per group; *bars*, SEM. **(D)** Representative fluorescent periaortic lymph nodes from animals treated with IgG control, docetaxel, anti-S1P mAb, anti-S1P mAb prior to docetaxel and combination of docetaxel and anti-S1P mAb, at the time of autopsy. Green arrows indicate prostate. Numbers represent periaortic lymph node volume as quantified in materials and methods. **(E)** Quantification of periaortic lymph nodes. *Columns,* mean of three to seven mice per group; *bars*, SEM.

Hypoxia plays a central role in tumor resistance to therapy including chemotherapy [45] and preclinical and clinical data support and offer a rationale of why a therapy aimed at increasing blood flow should result in improved drug delivery [39, 40]. We hypothesized that the vascular normalization period we observed with the anti-S1P mAb (optimal time of 5 to 9 days) offers an opportunity for enhanced response to docetaxel, the standard of care for the treatment of metastatic prostate cancer [61, 62]. To this end, we relied on an orthotopic model of PC-3 cells overexpressing GFP, growing in their native milieu and leading to a local regional growth and spontaneous distant metastasis dissemination within five weeks that could be monitored by fluorescence imaging [56]. The therapeutic relevance of anti-S1P mAb-induced increase in transient vascular normalization (or oxygenation window) to docetaxel was investigated by varying the sequence of anti-S1P administration and chemotherapy (Figure 6A).

In the first therapeutic approach, tumor-bearing mice were treated with anti-S1P mAb (50 mg/kg every other day) for a 5-day oxygenation window prior to 5 mg/kg docetaxel (Arm 2) or with a classical combination of anti-S1P mAb and docetaxel administered at the same time (Arm 3). Significantly smaller tumors (mean = 455 mm^3^) were seen in animals treated with 5 mg/kg docetaxel alone (Arm 5) (Figure 6B and 6C). Interestingly, administration of anti-S1P mAb alone (Arm 4) was not accompanied with a statistical reduction of primary tumor volume (mean = 724 mm^3^) as compared to IgG-treated control animal (Arm 1, mean = 1010 mm^3^) and the combination of S1P mAb with docetaxel administered at the same time (Arm 3, mean = 373 mm^3^) did not significantly sensitize to docetaxel alone (Arm 5) (Figure 6B and 6C). Clearly, the greater effect on primary tumor growth occurred when anti-S1P mAb was administered 5 days before docetaxel treatment (Arm 2, mean = 144 mm^3^). Supporting the notion that chemotherapy response could be optimum when docetaxel is given when after vascular normalization is achieved by anti-S1P mAb treatment, we demonstrated in Figure 6 that a significant difference (*P* = 0.0156) was observed between Arm 2 (anti-S1P mAb prior to docetaxel) and Arm 3 (anti-S1P mAb coincident with docetaxel) (Figure 6B and 6C). In addition to demonstrating an effect of antibody treatment on tumor volume, our fluorescent model allowed us to monitor metastatic dissemination in order to determine effects of the anti-S1P mAb on metastatic potential (Table 1). Although no treatment was able to significantly prevent lymph node tumor involvement (Table 1), we found a marked decreased in the volume of periaortic lymph nodes when anti-S1P mAb was combined to docetaxel (Figure 6D and 6E). As shown in Table 1, the anti-S1P mAb administration given before chemotherapy markedly reduced the total number of metastases (average per animal: 3.8) by comparison with isotype antibody control-treated Arm 1 (average per animal: 7.8). The effect of on primary tumor growth was thus consequently paralleled by a significant limitation of metastasis dissemination when anti-S1P mAb was given for 5 days before initiating chemotherapy.

**Table 1:** Pattern of metastatic dissemination

	IgG control	Docetaxel	anti-S1P	anti-S1P & Docetaxel	anti-S1P before Docetaxel
No. of mice with metastases/total No. of mice	**4/4** (*100%*)	**3/3** (*100%*)	**7/7** (*100%*)	**5/5** (*100%*)	**5/6** (*83%*)
Retroperitoneal lymph nodes					
Periaortic lymph nodes	2, 2, 2, 2	2, 2, 1	2, 2, 2, 2, 2, 2, 2	1, 2, 2, 2, 2	0, 2, 2, 2, 2, 2
Periadrenal	2, 2, 2, 1	2, 2, 2	2, 2, 2, 2, 1, 1, 1	1, 2, 1, 2, 2	0, 1, 1, 2, 1, 1
No. of metastases	**15/16** (*3.8 per animal*)	**11/12** (*3.7 per animal*)	**25/28** (*3.6 per animal*)	**17/20** (*3.4 per animal*)	**16/24** (*2.6 per animal*)
Solid organs					
Lung	1, 1, 1, 1	1, 1, 1	1, 1, 1, 1, 1, 1, 1	1, 1, 1, 1, 1	0, 1, 1, 1, 1, 1
Liver	1, 1, 1, 1	0, 1, 1	1, 1, 1, 1, 1, 0, 1	1, 1, 1, 0, 1	0, 0, 0, 1, 0, 0
Pancreas	1, 1, 1, 1	1, 1, 1	1, 1, 1, 1, 1, 1, 1	1, 1, 1, 0, 0	0, 0, 0, 0, 0, 0
Mesentery	1, 1, 1, 1	0, 1, 1	1, 1, 1, 1, 1, 1, 1	1, 1, 1, 0, 0	0, 0, 0, 1, 0, 0
No. of metastases	**16/16** (*4.0 per animal*)	**10/12** (*3.3 per animal*)	**27/28** (*3.9 per animal*)	**15/20** (*3.0 per animal*)	**7/24** (*1.2 per animal*)
Total No. of metastases	**31** (*7.8 per animal*)	**21** (*7.0 per animal*)	**52** (*7.4 per animal*)	**32** (*6.4 per animal*)	**23** (*3.8 per animal*)*

For retroperitoneal lymph nodes, the numbers 0, 1, or 2 represent the quantity of invaded lymph nodes. For solid organs, 0 means no presence of metastasis; 1 means a metastatic organ (regardless of the intensity of metastasis dissemination in this organ). The frequencies of metastases between two groups are not significant.

Differences in the number of metastases per mouse: *P = 0.0139 compared with IgG control-treated animals (Wilcoxon - Mann Whitney).

To rule out that improved therapeutic outcome caused by the sequential dosing (Arm 2) was not a mere reflection of the longer administration of anti-S1P mAb when compared to the classical combination treatment without pre-treatment (Arm 3), a second therapeutic approach was conducted with additional scheduling conditions (Supplementary Figure 1A). Treatments were initiated at Day 21 rather than Day 26 and compared to the sequential normalization treatment (Arm 2). Surprisingly, starting docetaxel treatment earlier did not improve the efficacy with regard to tumor growth (Arm 6 *versus* Arm 5) (Supplementary Figure 1B). When docetaxel was given as early as 21 days post-implantation in combination with anti-S1P mAb (Arm 8), there was no significant difference in terms of therapeutic efficacy compared to the 26-day conditions (Arm 3), implying that an extra 5-day duration of treatment did not matter (Supplementary Figure 1B). Importantly, the sequential scheduling (Arm 2) was always superior to all the others (Arm 3 or Arm 8) in terms of therapeutic efficacy (Supplementary Figure 1B).

In sum, these data suggest mechanistically that the anti-S1P mAb treatment leads to improved chemotherapy response when administered for a short period of time before the commencement of chemotherapy, as a consequence of antibody-induced vascular normalization.

In addition to efficacy studies described above, we conducted a toxicology study at the end of the experiment described in Figure 6 designed to evaluate how well the antibody was tolerated in mice. All biochemical and complete blood count (CBC) and serum chemistry markers were within normal range (Supplementary Figures 2 and 3). A statistically significant leukopenia was noted only in mice treated with the combination of anti-S1P mAb and docetaxel, which was mainly the consequence of a significant reduction in total circulating lymphocytes as reported previously [23, 29, 63].

## DISCUSSION

Hypoxia is a hallmark of solid tumors driving the production of angiogenic factors to establish a new vascular network [35, 37]. In contrast to healthy tissues, hypoxia-triggered overexpression of VEGF and other pro-angiogenic factors in tumors leads to a structurally and functionally abnormal vasculature, which further aggravates hypoxia setting up a vicious cycle promoting tumorigenesis and metastatic potential [36, 37]. Anatomically, tumor vessels are dilated, tortuous, and saccular with chaotic patterns of interconnection and branching [64]. Unlike a normal vasculature where stable endothelial cells are connected by adherens junctions including vascular endothelial (VE)-cadherin [65], VEGF causes VE-cadherin destabilization hence a loosening of endothelial cell association [66]. Moreover, pericytes, normally positioned around and interacting with endothelial cells to prevent vessel leakage are absent or loosely attached [67, 68]. The mechanisms for abnormal pericyte behavior in tumors are manifold, but include VEGF as a negative regulator [69, 70]. Taken as a whole, the structural weaknesses of tumor vessels, by contributing to a porousness vasculature phenotype, have adverse consequences notably dissemination of tumor cells [71, 72] and impaired drug delivery and efficacy [39, 40]. For these reasons, strategies aimed at restoring tumor vasculature to a more normal state are a matter of the utmost importance as the improvement of vessel structure and function may delay progression and improve delivery and efficacy of chemotherapeutics [41]. Because VEGF is primarily accountable for this haphazard vasculature, it was hypothesized by Rakesh Jain that mopping up excess of VEGF, rather than destroying vessels, would prune away some abnormal vessels and remodel the remaining ones resulting in a more mature vasculature [73, 74]. Over the years, a wide array of preclinical [75–77] and clinical [78–81] studies have provided evidence to support this postulate, showing that judicious treatment with anti-VEGF agents results in transient enhancement of vascular function termed “vascular normalization” characterized by improved connection between adjacent endothelial cells, increased ratio of pericyte-covered vessels, improved association between endothelial cells and pericytes, and reduced hypoxia [41]. Importantly, the accompanying period of enhanced oxygenation known as the “normalization window”, ranging in general from few days in mice models [41] to several weeks in some patients [81], corresponds to a period of enhanced chemo or radiosensitivity in treated tumors [40, 41, 75, 80]. Lastly, the most compelling support for the benefit of vascular normalization is its benefit on progression-free and overall survival rates in patients [81–84].

We recently identified the SphK1/S1P signaling as a new modulator of HIF-1α accumulation and increased activity under hypoxia, through activation of the Akt/GSK3β pathway [50, 85]. It was recently reported that direct addition of S1P to thyroid cancer cells stimulate Akt signaling under normoxic conditions [86]. Because Akt is activated by Gi-coupling of all subtypes of GPCR S1P receptors [10], and because S1P is secreted by hypoxic cells [51, 52], we analyzed the effects of the neutralization of extracellular S1P with the anti-S1P mAb, sphingomab [15]. Here we report for the first time that sphingomab inhibits HIF-1α accumulation and activity under hypoxia in various cell models (prostate, lung, glioma). This was a consequence of the secretion of S1P by hypoxic cells since knock-down of the S1P transporter Spns2 blocked the activation of HIF-1α, an effect that was reversed by the addition of S1P.

In an orthotopic prostate cancer animal model, we characterized the time course of morphological and functional changes in the vasculature of the tumor as well as intratumoral hypoxia in response to treatment with sphingomab. We demonstrated that S1P neutralization created a less hypoxic environment as shown by the marked decreased in HIF-1α expression and activity (concomitant decrease in GLUT-1 expression), and secretion of VEGF from the tumor. As a result, vascular remodelling occurred as demonstrated by decreased microvessel density and vessel morphological changes namely increased coverage of pericytes. As previously reported for anti-VEGF strategies in other preclinical models [75–77], the anti-S1P mAb was able to normalize intratumoral vasculature after a period (from 5 to 9 days) of treatment. Measurements of tumor blood flow and perfusion assessed by high resolution 3-dimensional power Doppler ultrasound [57, 58] demonstrated a transient improvement in tumor perfusion. Finally and most importantly, we established that the transient vascular normalization window induced by sphingomab created an optimal time frame for the administration of docetaxel-based chemotherapy with enhanced efficacy. Indeed, the therapeutic relevance of sphingomab-induced increases in tumor oxygenation to chemotherapy was investigated by varying the schedules of sphingomab and chemotherapy. Clearly, a greater benefit on both primary tumor and metastasis dissemination took place when sphingomab was administered 5 days prior docetaxel treatment.

Interestingly S1P has originally been shown to stabilize blood vessels in development and homeostasis through S1P receptor subtype 1 (S1P_1_) by promoting the formation of VE-cadherin-containing adherens junctions in endothelial cells [21, 87, 88]. Supporting this notion, the loss S1P_1_ in retinal endothelial cells results in vessels displaying poor blood flow and vascular leakage [89]. In addition, vessel coverage by pericytes is also directed by the activity of S1P_1_ receptor in endothelial cells [90, 91] by trafficking and activating N-cadherin involved in endothelial cell/pericyte interactions [92]. Our findings that neutralization of exogenous S1P with sphingomab leads to vascular maturation in hypoxic tumors are somehow counterintuitive to the undisputed role of S1P_1_, one of its main cognate receptor. Surprisingly it has been reported that S1P could increase vascular permeability similar to VEGF, the canonical vascular permeability factor [93]. The mechanism of action would involve the activation of the S1P_2_ subtype S1P receptor. This observation was extended to *in vivo* model of vascular permeability in the rat lung, in which the S1P_2_ antagonist JTE013 significantly inhibited H_2_O_2_-induced permeability [93]. *In vitro* and *in vivo* models of inflammation-induced vascular permeability confirmed the role of S1P_2_ [94]. These studies indicate that the relative expression of S1P_1_ and S1P_2_ receptors in a specific vascular bed would determine the response to S1P. Noteworthy, *in vivo* studies have shown that S1P_2_ expression is markedly enhanced under hypoxic stress in pathological angiogenesis of the mouse retina, establishing its essential role in pathological neovascularization [95]. Further supporting a critical role for S1P_2_ signaling in mediating vascular permeability, the addition of exogenous S1P to normoxic endothelial cells also induce the activation of HIF-1α and subsequent rise in VEGF release, suggesting a potential amplification of VEGF signaling [96]. Lastly, S1P_2_-deficient (S1p2−/−) mice implanted with lung or melanoma cells displayed increased number of maturated and functional tumor vessels, showing increased pericyte coverage [97]. In summary, one might speculate that the balance between S1P_1_ and S1P_2_ receptors in the endothelium could be modified by hypoxia, with a shift toward higher S1P_2_ expression hence increasing vascular permeability. Neutralization of S1P produced by hypoxic tumor cells by sphingomab could have a direct impact on vessels by switching off S1P_2_-mediated vascular leakage, a mechanism reminiscent of the effect of bevacizumab on VEGF. In addition, neutralization of S1P through inhibition of HIF-1α could impact the cancer cell compartment by reducing intratumoral hypoxia and its undesirable consequences (including VEGF overproduction), while sensitizing tumor cells to cytotoxic agents as S1P is a well-establish anti-apoptotic agent [5].

The extent of intratumoral hypoxia seen in the prostate cancer context is comparable to that seen in other cancers [98]. Since the seminal study conducted by Folkman's group showing that angiogenesis correlates with metastasis in invasive prostate cancer [99], numerous reports have confirmed an association between prostate cancer aggressiveness and increased microvessel density, greater irregularity of the vessel lumen and smaller vessels [100]. In a prostatectomy cohort of patients (*n* = 572) with clinically localized prostate cancer with 20 years of follow-up, men with the most irregularly shaped vessels were 17.1 times more likely to develop lethal disease several years after diagnosis [101]. These data clearly suggest that the morphologic characteristics that reflect the pattern and maturity of the growing vascular network could represent valid indicators of neoangiogenesis, cancer aggressiveness, and metastatic potential in prostate. In patients with metastatic castration-resistant prostate cancer, VEGF levels are independent predictors of overall survival [102]. These data supported the hypothesis that VEGF inhibition may enhance current therapies in metastatic prostate cancer. However, the failure of two recently published phase III trials [103, 104] based on combination regimens associating docetaxel with anti-VEGF strategies (bevacizumab or aflibercept) underline our limited understanding of the therapeutic targets being explored and poor preclinical investigation. No preclinical data to lend support to the combination of docetaxel and anti-VEGF approaches have been reported in the literature, and the vast majority of phase II trials have used non-randomized designs [105]. Despite the fact that these large trials have produced disappointing results with docetaxel-based combined anti-VEGF therapies, the notion that reduction of hypoxia and improvement of functional vascularization could sensitize to a therapeutic regimen has been successfully suggested in prostate cancer. Evidence exists that diminution of intratumoral hypoxia [106, 107], reduction of VEGF production [108] and improved functional vascularization [109] during androgen deprivation (ADT) would be accountable for the improved clinical outcome and patient survival observed in phase III clinical trials of short-term neoadjuvant ADT before radiotherapy [110, 111].

In view of these data supporting that only a neoadjuvant approach (ADT) could successfully sensitize to a therapy in prostate cancer, our preclinical findings may have clinical implications. Improvement in blood perfusion leading to better drug delivery or increased tumor oxygenation offers a rationale as to why S1P neutralization might perhaps be started prior to cytotoxic therapy (chemo- or radiotherapy) and continued through this therapy. Non-invasive imaging techniques are currently available in the clinic that could be used to assess tumor vascularity (i.e. DCE MRI) and/or oxygenation such as PET scanning with hypoxia sensitive tracers [79, 81, 82, 84, 109]. Hence, our findings encourage further clinical studies to assess whether optimized scheduling of anti-S1P treatment provide evidence for vessel normalization in cancer patients to identify those who might be benefit from a combination therapy with cytotoxic agents or radiotherapy.

## MATERIALS AND METHODS

### Chemicals, reagents and kits

Culture medium and serum were from Invitrogen (Villebon sur Yvette, France). The murine monoclonal anti-S1P antibody, Sphingomab, and its isotype-matched mAb control, LT1017, were generated as described previously [29]. S1P was from Avanti Polar Lipids (Alabaster, AL). Docetaxel was purchased from Sigma-Aldrich (St. Louis, MO). hVEGF plasma levels were quantified using a human VEGF Quantikine ELISA kit (RαD systems, Lille, France) according to manufacturer's instructions, respectively.

### Cell lines

Human prostate cancer PC-3 and lung carcinoma A549 cell lines were obtained from DSMZ (Braunschweig, Germany). Human U87 glioblastoma cells were from ATCC-LGC Standards (Molsheim, France). Cells were cultured in RPMI 1640 containing 10% fetal bovine serum at 37°C in 5% CO_2_ humidified incubators. Cell lines were routinely verified by the following tests: morphology microscopic examination, growth curve analysis and mycoplasma detection (MycoAlert™, Lonza, Basel, Switzerland). All experiments were started with low-passaged cells (<15 times). Hypoxia (0.1% O_2_, 5% CO_2_, 94.5% N_2_) was achieved using an In Vivo_2_ hypoxic workstation (Ruskinn Technologies, Bridgend, UK).

### siRNA-mediated knockdown of Spns2

Cancer cells were transfected with 90nM of each specific MISSION predesigned small interfering RNA (siRNA, Sigma-Aldrich) using Lipofectamine 2000 reagent (Invitrogen) according to the manufacturer's instructions. Aleatory sequence scrambled siRNA was from Eurogentec (Angers, France). Details of each specific MISSION predesigned siRNAs are provided below: human Spns2 siRNAa: 5′-CGCUCAUGCUCUGCCCU UUdTdT-3′; and human Spns2 siRNAb: 5′-CACUCAUC CUCAUUCUGGUdTdT-3′.

### Western-blot analysis and antibodies

Mouse HIF-1α (BD Biosciences, Le Pont de Claix, France), rabbit anti-Spns2 (Sigma-Aldrich) were used as primary antibodies. Western blot assays were conducted according to the manufacturer's instructions. Proteins were visualized by enhanced chemiluminescence detection system (Pierce, Brebières, France) using antirabbit or antimouse horseradish peroxidase–conjugated IgG (Bio-Rad, Hercules, CA). Equal loading was confirmed by probing the blots with the mouse anti-tubulin antibody (Sigma-Aldrich, clone DM1A). Densitometry quantitation was determined using Image J software (NIH, Bethesda, MD).

### Animals

Male NMRI/Nu (nu/nu) 6-wk-old mice were obtained from Elevage Janvier (Le Genest Saint Isle, France). Mice were housed in a barrier facility of high-efficiency particulate air-filtered racks. At 7–8 wk of age, the animals were used in accordance with the principles and procedures outlined in Council Directive 86/809/EEC. The Institut Fédératif de Recherche Bio-médicale de Toulouse Animal Care and Use Committee approved all animal studies.

### Blood and plasma samples

Blood samples were analysed using the ABX Micros 60 Hematology Analyzer and analytical parameters were determined in plasma by routine laboratory methods using an autoanalyzer (Cobas Mira+), Plateforme phenotypage ANEXPLO (Toulouse, France).

### Immunohistochemistry and immunofluorescence

Staining was conducted on paraformaldehyde (PFA)-fixed and paraffin-embedded tissue using 5 μm sections. Intratumoral hypoxia was assessed using a commercially available Hypoxyprobe™-1 kit for the detection of tissue hypoxia (Hypoxyprobe Inc., Burlington, MA). Pimonidazole hydrochloride was given at a dose of 60 mg/kg in 0.9% saline via intraperitoneal injection one hour before euthanasia, and orthotopic tumors were harvested and fixed in 4% PFA. Details regarding antibodies, dilutions, and antigen-retrieval methods used are provided in Supplementary Table S1.

### Orthotopic implantation of PC-3/GFP prostate cancer cells, and *in vivo* fluorescence imaging

Intraprostatic human prostate cancer xenografts were established in nude mice by surgical orthotopic implantation. Mice were anesthetized by isoflurane inhalation and placed in the supine position. A lower midline abdominal incision was made, and a 20 μL tumor cell suspension (1 × 10^6^ cells) was injected into the dorsal lobe of the prostate using a 30-gauge needle and glass syringe. The surgical wound was closed in 2 layers with 4–0 Dexon interrupted sutures. All procedures were performed with a dissecting microscope. Autopsy and *in vivo* fluorescence imaging were conducted as previously detailed [56].

### Tumor vessel perfusion by contrast-enhanced ultrasound imaging

Optical and ultrasound/power Doppler were carried out under anaesthesia (2% isofluorane in oxygen). Microbubble contrast agents were used to enhance *in vivo* visualization of microvascular perfusion of the tumors. This was operated with a Vevo 2100 linear array based high-frequency ultrasound imaging system (VisualSonics, Amsterdam, The Netherlands) and subsequent release of Nonlinear Contrast Imaging Mode. This allowed to assess the tumoral responses to anti-angiogenic drugs, even in absence of change in tumor size. The Micromarker Contrast Agent was delivered as a bolus injection intravenously (tail), and a time *versus* intensity curve is generated. The perfusion curve kinetics is an exponential limited by a maximum. The initial slope is proportional to the flow velocity and the maximum value of the plateau is proportional to the vascular fractional volume. The product of these two values gives a measure of the true perfusion. The perfusion model is defined as:

$$f(t)=O+A\frac{1}{st\sqrt{2\pi }}e\frac{{\left(\ln(t)-m\right)}^{2}}{2s^{2}},t>0$$

O, A, m and s are fitting parameters; O is the offset, A is an amplitude parameter and m and s are the mean and standard deviation of the normally distributed natural logarithm of t, respectively. Amplitude is expressed relative to echo power, time is expressed in seconds and both are combined to produce quantities relating to blood flow kinetics.

After infusion and steady-state perfusion, a destructive pulse could also be applied and a replenishment curve was generated.

The perfusion model is defined as:

$$f(t)=O+\frac{A}{2}\left[1+erf\left(\frac{\ln(t)-m}{s\sqrt{2}}\right)\right],t>0$$

where O, A, m and s are fitting parameters, as defined for equation 1.

### Image acquisition and processing and quantification

For bright-field and fluorescence, slides were scanned with Nanozoomer 2.0 RS Hamamatsu (with the fluorescence imaging module for tumor slides stained with anti-CD34, anti-αSMA, anti-cleaved-caspase-3, or anti-Ki67). Absolute numbers of CD34-positive vessels present within 1.5 mm^2^ of the tumor area and percent of αSMA/CD34-positive staining per 0.76 mm^2^ area and number of HIF-1α-positive cells per 0.8 mm^2^ area for each tumor slide were quantified by optical counting. Automatic cell counts of Ki67-positive and cleaved caspase-3-positive cells and automatic GLUT-1 and pimonidazole intensity were determined per 1.5 mm^2^ of the tumor area with Image J Software (NIH, Bethesda, MD).

### Statistical analysis

The statistical significance of differences between the means of two groups was evaluated by unpaired Student's *t* test. The frequencies of metastases between two groups were compared using Fisher's exact test. Differences in the number of metastases per mouse were examined using a nonparametric Wilcoxon - Mann Whitney test. All statistical tests were two-sided and the level of significance was set at *P* < 0.05. Calculations were done using Instat (GraphPad Software, San Diego, CA).

## SUPPLEMENTARY FIGURES AND TABLE


