# Supplementary material for: Neutralizing S1P inhibits intratumoral hypoxia, induces vascular remodelling and sensitizes to chemotherapy in prostate cancer
Source: Oncotarget. 2015 Jan 29;6(15):13803–21. doi: 10.18632/oncotarget.3144 (PMC4537051; doi:10.18632/oncotarget.3144)
Supplement: Supplementary file 1 [file oncotarget-06-13803-s001.pdf]

SUPPLEMENTARY FIGURES AND TABLE

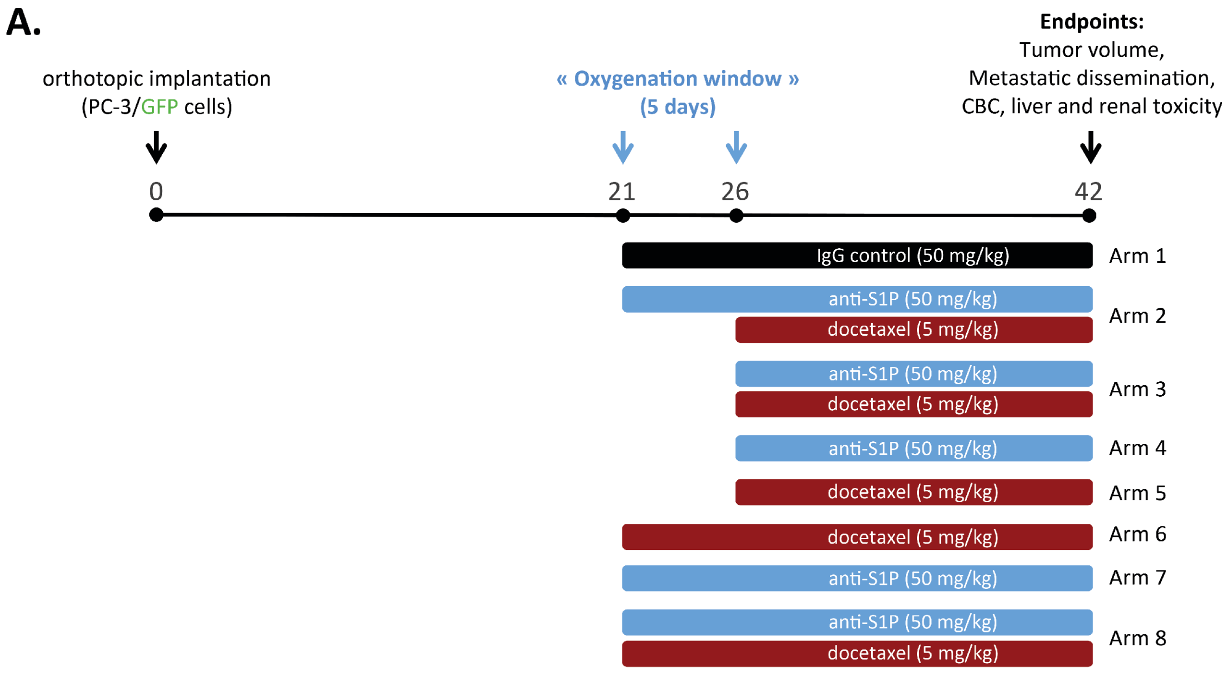

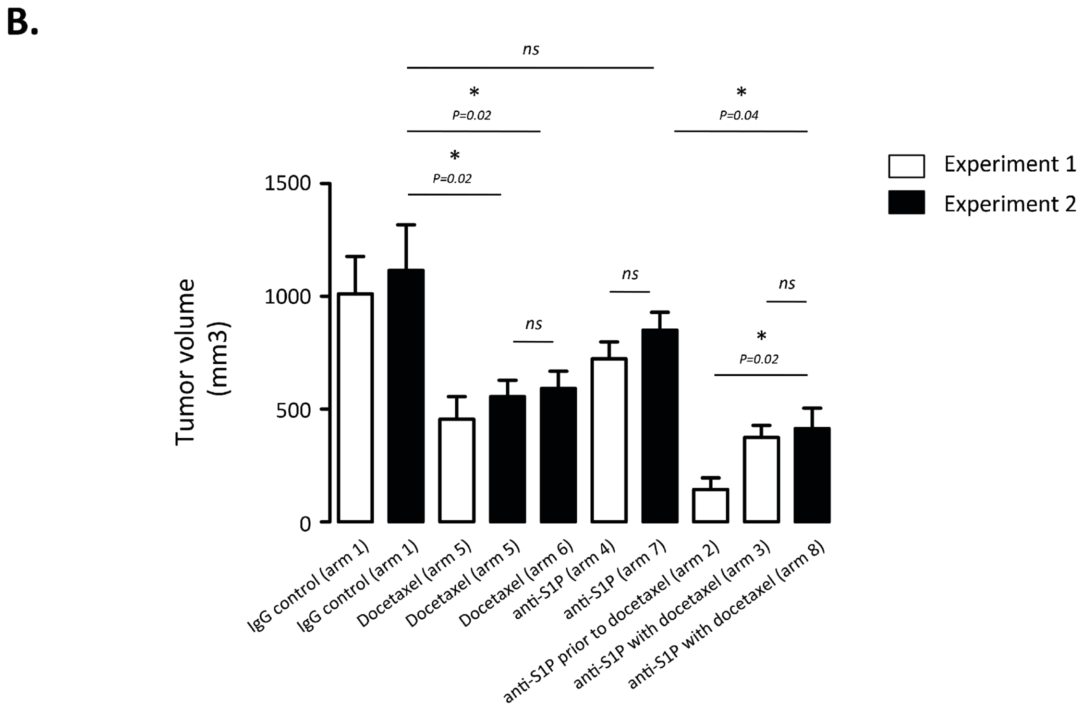

Supplementary Figure 1: (*Continued*)

	Experiment 1	Experiment 2
**Arm 1 :** 50 mg/kg IgG control every odd day starting at day 21	n=4	n=5
**Arm 2 :** 50 mg/kg anti-S1P mAb every odd day prior to weekly 5mg/kg docetaxel starting at day 26	n=6	
**Arm 3 :** 50 mg/kg anti-S1P mAb every odd day and weekly 5mg/kg docetaxel starting at day 26	n=5	
**Arm 4 :** 50 mg/kg anti-S1P mAb every odd day starting at day 26	n=7	
**Arm 5 :** weekly 5 mg/kg docetaxel starting at day 26	n=3	n=6
**Arm 6 :** weekly 5 mg/kg docetaxel starting at day 21		n=8
**Arm 7 :** 50 mg/kg anti-S1P mAb every odd day starting at day 21		n=4
**Arm 8 :** 50 mg/kg anti-S1P mAb every odd day and weekly 5mg/kg docetaxel starting at day 21		n=6

Supplementary Figure 1: Effects on primary tumor growth of various treatments using anti-S1P mAb and docetaxel. (A) Treatment scheduling in PC-3/GFP xenografted nude mice of two independent experiments. Three weeks after surgical orthotopic implantation of PC-3/GFP cells, mice were randomized into several arms of three to eight animals each as follows: **(B)** Quantification of tumor volume of primary tumors in Experiment #1 and #2. (*Columns*, mean of three to eight mice per group; *bars*, SEM).

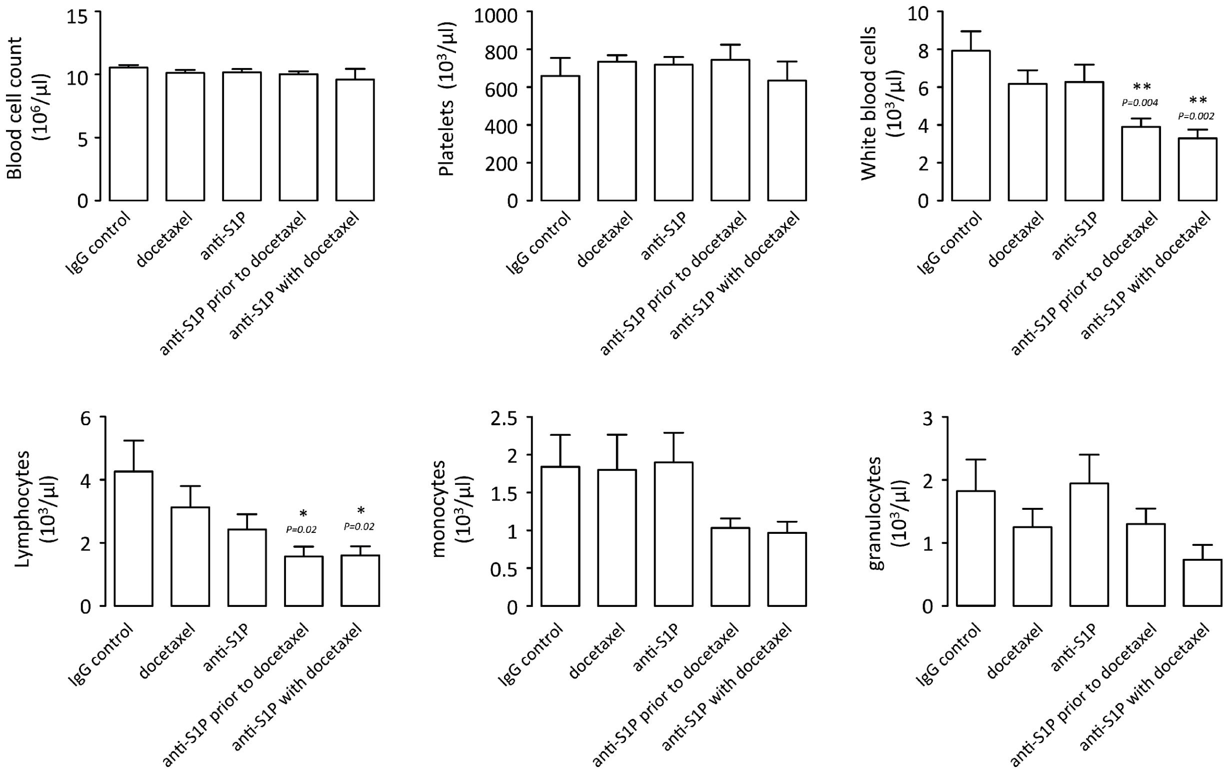

Supplementary Figure 2: Effect of treatments on Complete Blood Count. Blood cell count, platelets, white blood cells, lymphocytes, monocytes and granulocytes in plasma concentrations in tumor-bearing mice at the end of the protocol after treatment with IgG control (arm 1), docetaxel (arm 5), anti-S1P mAb(arm 4), anti-S1P mAb prior to docetaxel (arm 2) or combination of anti-S1P mAb and docetaxel (arm 3). *Columns*, mean of four to seven mice per group; *bars*, SEM. Blood samples were analysed using the ABX Micros 60 Haematology Analyzer.

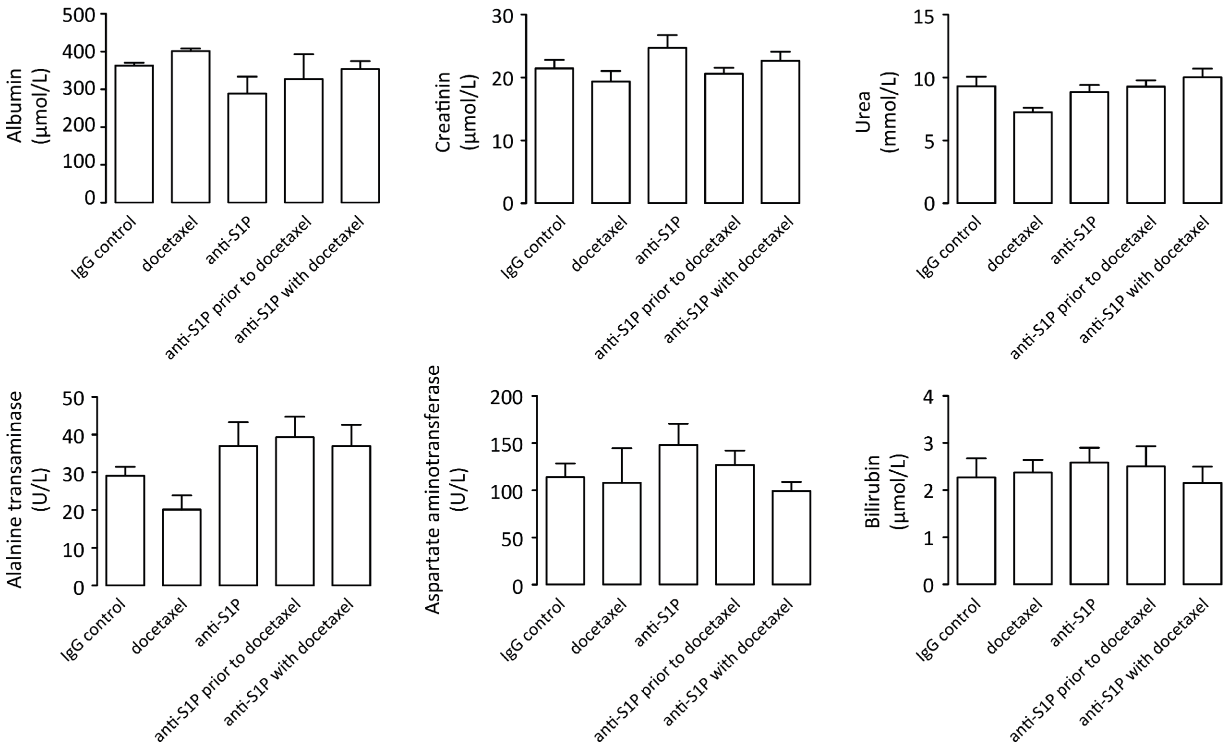

Supplementary Figure 3: Effet of treatments on serum chemistry. Albumin, Creatinin, Urea, Alanine transaminase, Asparate aminotransferase and bilirubin in plasma concentrations in tumor-bearing mice at the end of the protocol after treatment with IgG control (arm 1), docetaxel (arm 5), anti-S1P mAb(arm 4), anti-S1P mAb prior to Docetaxel (arm 2) or combination of anti-S1P mAb and docetaxel (arm 3). *Columns*, mean of four to seven mice per group; *bars*, SEM. Analytical parameters were determined in plasma by routine laboratory methods using an autoanalyzer (Cobas Mira+; Plateforme phenotypage ANEXPLO).

Supplementary Table 1: List of antibodies used for immunohistochemistry studies

Antigen	Antibody	Dilution	Species	Retrieval method	Secondary Antibody
HIF-1α	Anti-HIF-1α[H1alpha67] (Abcam, ab1)	1/200	Mouse IgG	Citrate pH6.0	Vector M.O.M Immunodetection kit. PEROXIDASE (Vector Lab)
GLUT-1	GLUT-1 (Neomarkers, RB-9052)	1/200	Rabbit IgG	Citrate pH6.0	ImmPRESS REAGENT Anti-Rabbit Ig(Vector Lab)
CD34	Anti-CD34 (MEC 14.7) (Abcam, ab8158)	1/400	Rat IgG	Citrate pH6.0	Anti-Rat Ig biotinylated (DAKO) and Cy3 Streptavidin (Jackson Immunoresearch)
αSMA	Actin Smooth Muscle Clone 1A4 (DAKO, M0851)	1/75	Mouse IgG	Citrate pH6.0	Alexa Fluor 488 Affinity-Pure Donkey anti-mouse IgG (Jackson Immunoresearch)
